# Immuno-proteomics analysis between OMV of vaccine and dominant wild type strains of *Bordetella pertussis* in Iran

**Published:** 2020-04

**Authors:** Ali Badamchi, Fariborz Bahrami, Alireza Hadizadeh Tasbiti, Shamsi Yari, Morvarid Shafiei, Fereshteh Shahcheraghi, Seyed Davar Siadat

**Affiliations:** 1 Department of Bacteriology, Pasteur Institute of Iran, Tehran, Iran; 2 Department of Immunology, Pasteur Institute of Iran, Tehran, Iran; 3 Department of Tuberculosis and Pulmonary Research, Pasteur Institute of Iran, Tehran, Iran

**Keywords:** *Bordetella pertussis*, Outer membrane vesicles, Vaccine, Mass spectrometry analysis

## Abstract

**Background and Objectives:**

Despite widespread vaccination programs against pertussis, there has been a worldwide resurgence of the disease in recent years. We aimed to investigate protein composition of outer membrane vesicles (OMV) of *Bordetella pertussis (Bp)* and to evaluate the immunogenicity of OMV antigens both in the vaccine and the dominant wild type strains in Iran.

**Materials and Methods:**

The OMV were purified from both vaccine and wild type strains. The immunoreactivity of the OMVs was investigated by exposing sera taken from the patients and the vaccinated infants. The protein profiles of OMVs were compared using two-dimensional electrophoresis. The LC-MS/MS was used to analyse and identify differentially expressed protein spots.

**Results:**

The two type strains showed differences in their 2D gel protein profile. Further analysis of selected proteins from the dominant Iranian strains using LC-MS/MS demonstrated that the identified proteins fell into different functional categories including (i) metabolism, (ii) membrane transport and secretion system, (iii) biosynthesis and degradation, (iv) adaption, adhesion, pathogenicity, conserved hypothetical and protection responses. Moreover, a number of immunogenic proteins were identified including *Bp* 2434 (serine protease) and *Bp* 1616 (putative DNA binding protein) from the vaccine and the wild type strains, respectively which could be considered as potential antigens for an OMV vaccine.

**Conclusion:**

OMV *Bp* could be considered as an alternative vaccine against pertussis, containing the bacterium’s protein antigens that can confer equal efficacy compared to a whole bacterial cell vaccine with advantages such as less side effects and lower costs than acellular pertussis vaccines.

## INTRODUCTION

Pertussis, also known as whooping cough, is one of the main vaccine-preventable infectious diseases, caused by a coccobacillus bacterium, named *Bordetella pertussis (Bp)*. As estimated by World Health Organization (WHO), 50 million cases and 300’000 deaths are caused by pertussis every year (1). After 1940s, the public vaccination programs which were based on a formalin-killed whole bacterial cell vaccines (wP) reduced the incidence and the mortality rate in the developed countries (2). However, high fevers with or without febrile seizures, swelling, pain and redness at the site of injection have been reported as the most common side effects of wP vaccines (3). Later, acellular pertussis vaccines (aP) were developed in recent decades (4). Although aP vaccines are less reactogenic than wP vaccines, the duration of protection offered by aP vaccines has been reported to be shorter (5). Despite widespread vaccination programs by aP and wP vaccines, there has been a worldwide resurgence of pertussis in recent years and it remains a major cause of vaccine-preventable deaths, particularly in the developing countries (6). Moreover, the causes of pertussis outbreaks and its rising incidences in some countries including Iran, after 60 years of history with pertussis vaccination program, have remained poorly understood.

Outer membrane vesicles (OMVs) are spherical blebs with an average diameter of 20–300 nm that are naturally released from Gram-negative bacteria into the environment (7). OMVs of *Bp* (omvPV) are considered as a potential vaccine candidate against pertussis (8). An immune response with mixed Th1/Th2/Th17 lymphocytes has been detected in omvPV-mediated immunizations (8, 9). However, omvPV induces higher serum IgG antibody and neutrophil response with less pro-inflammatory cytokines compared to wP. On the other hand, omvPV stimulates both innate and adaptive immune responses (8) while it can improve efficacy in terms of vaccine reactogenicity, as observed in animal models studies (8). The question is whether omvPV can induce efficient immune responses against pertussis in humans. The aim of the present study was to elucidate the immunoproteomic differences between OMVs of a dominant *Bp* vaccine strain (named *Bp* 134), compared to a wild type *Bp* strain isolated from a patient in Iran (named *Bp* IP91), using qualitative proteomic analyses. Protein profiles were generated using a multi-combinatorial approach of SDS-PAGE, two-dimensional electrophoresis (2DE) and Liquid chromatography–mass spectrometry (LC-MS/MS) tryptic peptide detection and OMP identification via database search analysis. Western blot was used to identify *Bp* immunoreactive proteins. Our preliminary study revealed that six differentially-expressed proteins in OMV of the wild strains provide novel targets for further evaluation and development of an efficient regional vaccine against pertussis.

## MATERIAlS AND METHODS

### Bacterial strains.

*Bp* vaccine strain 134 (*Bp* 134) as well as the wild-type strain (*Bp* IP91) (10) were cultured on Bordet-Gengou (BG) agar medium (BG; Difco, USA), containing 15% sheep blood. *Bp* IP91 was selected as a representative predominant circulating strain in Iran. This strain was included in the cluster of predominant PFGE pattern and isolated in 2012 from a 4 months old female baby. *Bp* 134 was obtained from Department of human vaccine and serum, Razi vaccine and Serum Research Institute, Karaj, Iran. The genotype of the strains were as follows: *Bp* 134 (ptxS1B, Fim 3-1, prn1 and cyaA2) and *Bp* IP91 (ptxS1A, Fim 3-2, prn2 and cyaA2) (10).

### Purification and protein quantification of OMVs.

OMVs were prepared as previously described (9), with minor modifications. Briefly, *Bp* IP91 and *Bp* 134 strains were grown in Stainer Sholte (SS) broth at (36 ± 1 °C; 160 rpm) until they reached late log phase at 25 and 27 h, respectively. Heat-killed whole cells (56 °C for 30 min) of each strain were used in the experiments. Culture samples from the log phase were centrifuged at 10,000 × g for 20 min at 4 °C and the pellets were re-suspended in 20 mM Tris–HCl, 2 mM EDTA, pH 8.5 (TE buffer). The suspension was sonicated on ice for 10 min with a Dismembrator (Fisher). After two centrifugations (10,000 × g for 20 min at 4 °C), the supernatant was pelleted at 100,000 × g for 2 h at 4 °C. This pellet was re-suspended in 1.5% (w/v) deoxycholate (DOC) in TE buffer. Six ml of this suspension was added to 2 ml of an aquatic sucrose 60% (w/v) solution. After centrifugation at 100,000 × g for 2 h at 4 °C, the OMV bands were observed at TE/sucrose interphase and dialyzed overnight at 4 °C with 10 mM Tris HCl buffer (pH 8.0) with a 12-kDa cutoff dialysis tube (Sigma-Aldrich, Germany). To ensure that the supernatant was free of viable bacteria, 1 mL of the supernatant was streaked on BG agar and incubated at 37 °C for up to 72 h. The OMVs were stored in 1% glycerol and 0.001% sodium azide at 4 °C. OMVs of the strains were quantified by Bradford protein assay (Bio-Rad) using bovine serum albumin (BSA) as the standard (11).

### Transmission electron microscopy (TEM).

Electron microscopy was performed by suspending OMVs in 0.1 M ammonium acetate (pH 7.0). A droplet of this suspension was placed on a grid, coated with a 10 nm carbon-reinforced 10 nm Formvar film. After 30 s, the excess fluid was removed by absorbing with a filter paper and the grids were stained with 2% (w/v) phosphotungstic acid (pH 5.2 with KOH). Examination was done with a JEM 1200 EX Jeol microscope (Japan).

### Measurement of IgG-PT and IgA-PT in serum samples.

Serum samples were collected from 100 infants in two age groups including 35 samples from infants younger than two months of age and 65 samples from infants 7–12 months old. The samples were stored at −20 °C until use. The levels of IgGPT (Pertussis toxin) and IgA-PT -were measured using a commercial ELISA kit (IBL-Hamburg GmbH, Germany), with a manufacturer’s stated sensitivity of > 95% (12). All IgG and IgA antibody concentrations were expressed as geometric mean concentrations (GMCs). Informed consent was obtained from parents of all participants in this study. The Ethical Committee of Pasteur Institute of Iran approved this study (letter no 96.0201.20877).

### Western blot analysis of OMV protein content.

The OMV samples were subjected to Western blotting analysis. Briefly, 250 μl trichloroacetic acid (TCA) were added to 1.0 ml OMV content and incubated for 10 min at 4 °C. The tubes were spun down at 21,000 × g for 5 min. The pellet was washed three times with cold acetone and dried at room temperature (RT). Protein expression was checked using SDS-PAGE followed by either Coomassie staining or immunoblotting analysis. Samples were prepared by adding 5 × SDS loading dye, denatured at 95 °C for 5 min and centrifuged at 10000 × g for 1 min. The samples were separated on an SDS-PAGE (12.5%) gel. The gel was run at 160V for 1 h in Tris-Glycine SDS running buffer. Thirty five μg of proteins from OMV of *Bp* were run using (12.5%) SDS-PAGE (9) and the separated proteins were subsequently transferred to PVDF membranes within 15 h at 50 mA. The membranes were blocked with 1.5% BSA in TBST overnight at 4 °C (BioRad) and then washed twice with TBST. The membranes were incubated overnight at 4 °C with pooled sera from children who were vaccinated with Diphtheria-Tetanus-Pertussis (DTwP); and negative control sera as shown in the Table 1. Triplicate washing of the membranes with TBST was performed and then they were incubated with goat anti-mouse HRP-conjugated secondary antibody (1:1000–1:2000 dilutions; Thermo Scientific Pierce, USA) for 2 h at laboratory temperature. The membranes were subsequently double-washed with TBST and finally developed with DAB (3, 3′-Diaminobenzidine) substrate (Millipore Sigma, USA).

**Table 1.** Classification of sera samples into different groups

**Control sera**	**patient (n=4)**	**Vaccinated (n=16)**	**Negative (n=16)**
Clinical symptoms	Yes	No	No
Vaccination status	No	Yes	No
IgG-PT (IU^1^/ml)	(52.7 ± 18.8)^2^	(94.8 ± 12.8)	(2.2 ± 3.5)
IgA-PT (IU/ml)	(23.9 ± 1)	(2.6 ± 1.5)	(1.1 ± 2)

1 IU, international unit;

2 (mean ± SD).

### Two dimensional gel electrophoresis (2DE).

2DE was performed using the Ettan IPGphor 3 isoelecteric focusing system (GE Healthcare, USA). The incubation of the samples for 2DE was performed after sample preparation and IR-labeling. About 350 μg OMV *Bp* proteins was incubated overnight at laboratory temperature in DeStreak Rehydration solution (final volume = 250 μL) which contained 7 M urea, 2 M thiourea, 100 mM, 4% CHAPS, 0.2% carrier ampholyte, 0.0002% bromophenol blue, 4 mg of 50 mM dithiothreitol (DTT) and 1.5 μL of IPG buffer (pH 3-10 NL) (GE Healthcare). Immobiline Dry Strips pH 3-10 NL, 7 cm (GE Healthcare) were rehydrated with protein sample in an IPGbox (GE Health-care). Isoelectric focusing (IEF) was performed in an Ettan IPGphor 3 IEF system (GE Healthcare) according to the following conditions: 300 V for 30 min, 1000 V for 30 min, 5000 V for 90 min, finally 5000 V for 8 KVh, at 20 °C and a current limit of 50 μA per strip. After IEF, strips were equilibrated in 3 mL of equilibration buffer (150 mM Tris-HCl, pH 8.8, 6 M urea, 50% glycerol, 20% SDS, and bromophenol blue) with DTT (2%) for 15 min at RT, followed by 3 mL of equilibration buffer with iodoacetamide (2.5%) for 15 min at RT. The proteins were separated in second dimension on 10% SDS -PAGE in a vertical electrophoretic dual gel system and were visualized by Coomassie (R-250) staining method (13).

### Mass spectrometry procedure.

Protein bands were sent to PhenoSwitch Bioscience Canada for mass spectroscopy, briefly, sample preparation gel bands were dehydrated in 50 mM tris pH 8.0 + 50% acetonitrile and rehydrated with 10mM DTT for 15’ at 65 °C. Proteins were then alkylated with 15 mM iodoacetamide for 30 minutes in the dark, at RT. Gel bands were dehydrated again to remove excess re-agents and were rehydrated in 50 mM tris pH 8.0 + 1 μg of Trypsin/LysC. The digestion was carried over night at 37 °C with agitation. Peptides were extracted from the gel by dehydratation with 50% acetonitrile in 5% formic acid for 30 min, followed twice. Acetonitrile was evaporated by speedvac and the remaining peptides were purified by reversed phase solid phase extraction before LC-MS/MS analysis. Acquisition was performed with an ABSciex TripleTOF 5600 (ABSciex, Foster City, CA, USA) apparatus. The electrospray interface with a 25 μm i.d. capillary was used and coupled to an Eksigent μUHPLC (Eksigent, Redwood City, CA, USA). Analyst TF 1.7 software was used to control the instrument and for data processing and acquisition. Acquisition was performed in Information Dependant Acquisition (IDA) mode. A 5.2 kV source voltage was set and maintained at 225 °C, curtain gas was adjusted at 27 psi (the first gas; 12 psi and the second; 10 psi). A reversed phase HALO C18-ES column 0.3 μm i.d., 2.7 μm particles, 50 mm long (Advance Materials Technology, Wilmington, DE) which was maintained at 50 °C was used for separation. Samples were injected by loop overfilling into a 5 μl loop. For the 5 minutes (IDA) LC gradient, the mobile phase consisted of the following: solvent A (0.2% v/v formic acid and 3% DMSO v/v in water) and solvent B (0.2% v/v formic acid and 3% DMSO in EtOH) at a flow rate of 10 μl/min.

### Data analysis.

All runs were analyzed simultaneously with Protein Pilot ^TM^ v4.0 software (14). In addition to spot identification by PMF, a detailed analysis of these identified immunoreactive proteins was performed using a variety of bioinformatics tools. A modified version of a previously developed approach was used to predict proteins localization at the sub-cellular scale. The Pfam (protein families database; http://www.sanger.ac.uk/Software/Pfam/) was used for such identifications and the pI/MW tool which is available at http://www.expasy.org/tools/pi_tool.html/) was used for estimation of the theoretical molecular weights and the isoelectric points.

## RESUlTS

### Characterization of OMVS using TEM.

**Fig. 1.**
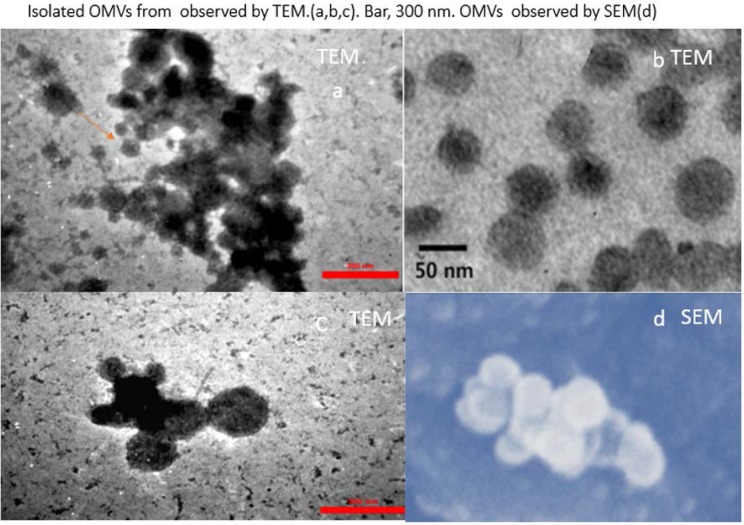
TEM and SEM images of negatively-stained OMVs, extracted from *Bp*. Bar: 300 nm.

OMVs with a mean size of 80 nm ranging from 20 to 250 nm were obtained from *Bp* IP91 and *Bp* 134 strains (Fig. 1) which were compatible with the size ranges of OMVs in previous studies (15).

### Serum samples were classified into three control groups.

The mean age of the study population was six months. The GMCs of anti-PT and anti-o antibodies were 18.8 and 17.9 EU/ml, respectively. The average titer for IgG-PT and IgA-PT (IU/ml) were 40.43 ± 33.02 and 6.23 ± 5.3 (Mean ± SD), respectively. The infants were divided into two groups, namely; less than 2 months of age and 7–12 months old. Among 100 cases enrolled in this study, 35 infants were younger than 2 months of age and 65 cases were between 7–12 months. Members of the former group were not vaccinated, although the participants in the second group were vaccinated by DTwP. The antibody titration results showed that 11.4 (4/35) and 11.4 (4/35) percent of the infants < 2 months were positive for IgA-PT and IgG-PT, respectively. On the other hand, the frequencies of the infants aging 7–12 months were 7.7 (5/65) and 76.9 (50/65) percent for IgA and IgG titers, respectively.

In the next step, the sera were analyzed with respect to the positivity or negativity for IgA and IgG, clinical symptoms and the infant’s vaccination status, in order to select the control groups. As shown in Table 1, the samples were classified into three groups, including a patients group which were not vaccinated, positive for IgA and IgG and with clinical symptoms, a vaccinated group who were positive for IgG (and negative for IgA) and a negative control group who were negative for all the selection criteria. According to Table 1, 36 classified serum samples were selected for Western blot analysis. The mean anti-PT titer was higher in the vaccinated group, compared with the patients group. Meanwhile, the mean IgA titer was elevated in the patients group, indicating the nature of the disease.

### OMV’s of the wild type strain were more immuno-reactive than the vaccine strain.

**Fig. 2.**
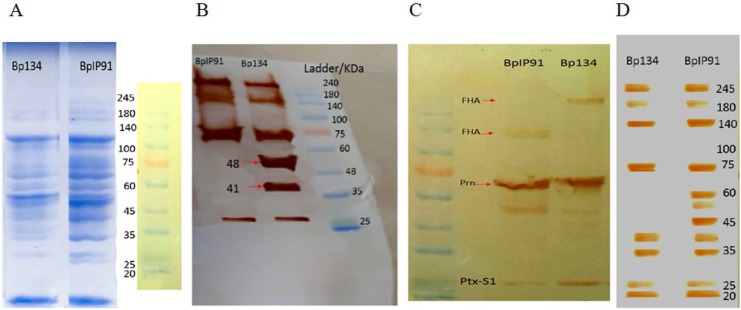
Protein profiling of OMVs of *Bp*. (A) 25 mg OMV *Bp* 134 and OMV *Bp* IP91 separated by SDS-PAGE. The gels were stained by Coomassie. (B) Immunoblotting was performed on the serum samples (1:200 dilution), obtained from the vaccinated children. (C) Immunoblotting was performed using monoclonal Antibodies (FHA 1:2000, PRN 3:10000, Ptx 3:1000). The presence of pre-FHA and pre-PRN is observed as double bands for each antigen. (D) Immunoblotting was performed using serum samples from 4 patients (1:100 dilution).

According to SDS-PAGE results (Fig. 2A), a similar pattern was illustrated for OMV *Bp* 134 and OMV *Bp* IP91 strains. Further experiments, using Western immunoblotting with the serum samples indicated that OMVs of the vaccine strain possess bands with 41 and 48 KDa molecular weights which were absent in OMV of the wild type strain (Fig. 2B). In order to investigate whether the observed difference in serum immune-blotting was due to some of the main antigens included in aP vaccines, immunoblotting was performed using monoclonal antibodies against FHA, Prn and Ptx antigens. As speculated for the Fig. 2C, the observed differences in serum-immune blot result could not be related to these antigens. This further emphasized the presence of those antigens in OMV’s of both strains. In order to discriminate the reactivity of the patients sera compared with the vaccinated infants against the vaccine and wild type strains, a separate set of experiments was conducted using OMV strains incubated with the patients pooled sera. As depicted in Fig. 2D, the patients pooled sera interacted with the wild type strain which revealed more immunogenic bands, compared with the vaccine strain. These results may be indicative of the fact that OMVs of the wild type strain express more immuno-reactive antigens than the vaccine strain.

### Proteomics and differential expression of proteins.

**Fig. 3.**
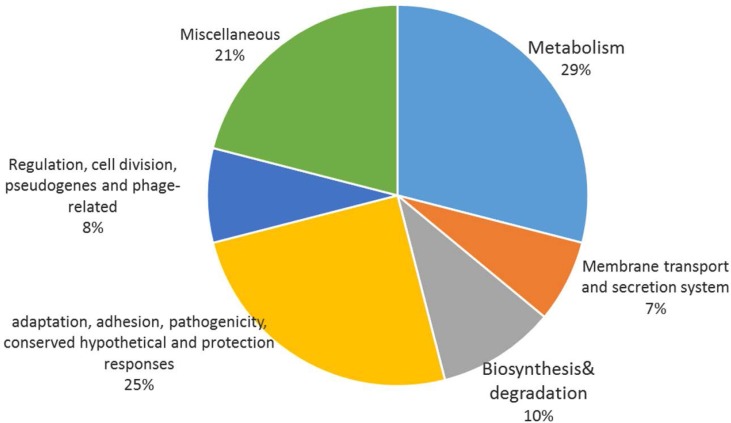
Functional classes of proteins identified from OMV of *Bp.*

**Fig. 4.**
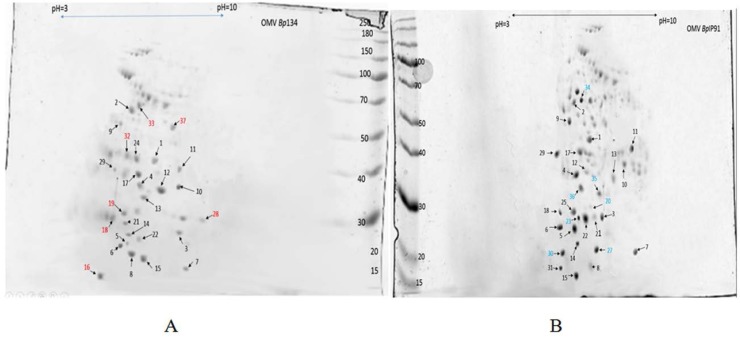
Protein fingerprint of OMV from *Bp* both strains by 2-D gel electrophoresis. Total protein lysates (350 μg total protein) of OMV *Bp* 134 (A) and OMV *Bp* IP91 (B) were separated by 2-D gel electrophoresis. Spots are indicated by arrows. The gels were stained by Coomassie blue and then were excised and analyzed by LC-MS/MS. Different colors correspond to proteins that are present in both OMVs (Black), only present in OMV *Bp* 134 (Red), and only present in OMV *Bp* IP91 (Blue). For each OMV content profile, three technical replicates were characterized and one representative gel is presented.

The differences between OMV *Bp* 134 and OMV *Bp* IP91 groups are shown in Fig. 3. The significant differences between 2-DE images of OMV *Bp* 134 and OMV *Bp* IP91 were observed. All the spots were matched by a gel-to-gel comparison and the relative abundance difference (volume percent) of each spot was determined. The spots which had abundance at least significantly different by ± 2 fold in OMV *Bp* IP91 versus OMV *Bp* 134 were chosen for identification. The protein spots which were vigorous to other spots to enable them to be picked up with confidence were chosen. Using these criteria, 40 spots statistically confirmed protein variations between OMV *Bp* 134, OMV *Bp* IP91 were selected. In total, 37 proteins were successfully identified and marked on the gel as shown in Fig. 4. The results obtained from recognized proteins and their functions are summarized in Table 2.

As shown in Fig. 3, after annotation of the functional classes of a total of 37 identified proteins, they fell into 6 categories as follows: (i) metabolism (29%), (ii) membrane transport and secretion system (7%), (iii) biosynthesis and degradation (10%), (iv) adaptaion, adhesion, pathogenicity, conserved hypothetical and protection responses (25%), (v) regulation, cell division, pseudogenes and phage-related (8%), and (vi) Miscellaneus (21%). It was found that the majority of identified proteins in OMV *Bp* 134 was down-regulated. The proteins including Lipoprotein (spot no. 3) and Ribulose-phosphate 3-epimerase (spot no. 4) were down-regulated proteins in OMV *Bp* 134. Alkyl hydroperoxide reductase (AhpC; spot no. 8) was overexpressed in the OMV *Bp* 134. The role of AhpC in detoxification of reactive oxygen species has been shown in previous studies.

While a number of protein spots were common among OMV of the vaccine strain and OMV of *B. pertussis* IP91. There were multiple spots unique to *B. pertussis* IP91, i.e., Dihydrolipoyl dehydrogenase (spot 34; BP0995), Putative DNA-binding protein (spot 27; BP1616), Inorganic pyrophosphatase (spot 30; BP2533), a Putative enoyl-CoA hydratase (spot 25; BP3277). Five of protein spots were common among OMV of the vaccine strain tested and OMV of *Bp* IP91 including (BP2759), (BP2377), (BP2818), (BP3266) (BP0965), (BP0205) and BP0558. (Figs. 4A and B).

**Table 2.** Protein composition of OMV*Bp*134 and OMV *Bp* IP91determined by LC–MS

**Protein No.^a^**	**Gene**	**%Cov**	**Name**	**MW**	**PI**	**Length(aa)**	**Subcellular localisation**
1	BP2759	2.743	Putative thiolase	41.473	6.8	401	periplasm
2	BP2377	1.243	Putative AMP-binding protein	62.3	5.8	563	cytoplasm
3	BP2818	26.04	Lipoprotein	28.7	7.7	265	cytoplasm
4	BP0182	6.135	Iron-sulfur cluster carrier protein	38.2	6.2	163	cytoplasm
5	BP3266	4.898	Ribulose-phosphate 3-epimerase	26.426	5.8	245	cytoplasm
6	BP0965	28.64	Antioxidant protein	23.777	5.6	213	cytoplasm
7	BP0205	34.04	Putative exported protein	11.77	8.4	114	Extracellular
8	ahpC	23.08	Alkyl hydroperoxide reductase	18.38	5.1	172	cytoplasm
9	glcD	3.206	Glycolate oxidase subunit	53.2	5.29	499	cytoplasm
10	sbp	60.47	Sulfate-binding protein	37.8	7.79	344	Outer membrane
11	smoM	21.7	Putative periplasmic solute-binding protein	40.01	7.36	364	cytoplasm
12	BP0558	14.79	Amino acid-binding periplasmic protein	36.1	6.6	338	periplasm
13	BP3809	20.31	Uncharacterized protein	34.1	6.23	320	cytoplasm
14	BP2891	13.79	Uncharacterized protein	22.641	6	203	cytoplasm
15	BP3465	4.167	Uncharacterized protein	18.809	6.2	168	cytoplasm
16	BP2782	32.88	Lipoprotein	8.056	4.7	73	Outer membrane
17	adhI	9.239	S-(hydroxymethyl)glutathione dehydrogenase	39.4	6.2	368	periplasm
18	minD	60.89	Site-determining protein	29.6	5.2	271	cytoplasm
19	BP0103	59.2	Probable class IV aminotransferase	32.2	5.4	299	cytoplasm
20	sdhB	31.93	Succinate dehydrogenase iron-sulfur subunit	27.2	6	238	cytoplasm
21	BP3757	17.27	Putative ABC transporter, ATPbinding protein	29.6	5.2	278	cytoplasm
22	BP1300	44.21	Putative glutathione transferase	26.4	6.2	233	cytoplasm
23	BP0961	9.639	Electron transfer flavoprotein beta subunit	26.819	5.7	249	cytoplasm, periplasm
						340	
24	BP2963	5.294	Putative membrane protein	40.055	7.6		Outer membrane
25	BP1702	3.409	Probable enoyl-CoA hydratase/isomerase	28.556	5.7	261	cytoplasm
26	odhB	1.98	oxoglutarate dehydrogenase complex	41.825	5.3	404	cytoplasm
27	BP1616	27.71	Putative DNA-binding protein	18.52	6.2	166	cytoplasm
28	BP3550	2.545	AraC-family regulatory protein	30.6	9	275	cytoplasm
29	bopN	1.918	Putative outer protein N	38.99	5	365	cytoplasm
30	BP2533	46.07	Inorganic pyrophosphatase	20.042	5.3	178	cytoplasm
31	BP0361	25.79	Uncharacterized protein	16.88	5.2	159	Outer membrane
32	gltA	13.99	Citrate synthase	48.46	6.03	436	cytoplasm
33	sdhA	4.899	Succinate dehydrogenase flavoprotein subunit	64.8	6.1	596	cytoplasm
34	lpdA	5.872	Dihydrolipoyl dehydrogenase	62.3	6.1	596	cytoplasm
35	BP3277	45.45	Putative enoyl-CoA hydratase	30.1	6.2	275	cytoplasm
36	mmsB	19.19	3-hydroxyisobutyrate dehydrogenase	29.6	5.7	297	cytoplasm
37	BP2434	2.828	Periplasmic serine endoprotease DegPlike	52.1	7.6	495	periplasm, Outer membrane

Identifications were confirmed by LC- MS/MS ion search and significant matches (p < 0.05) were retained (Swiss-Prot protein database).

a: Protein numbering refers to spot numbers in Fig. 4.

## DISCUSSION

*Bordetella pertissis* is an important pathogen during childhood that causes pertussis. The protective effectiveness of present wP vaccines is not satisfactory. Meanwhile, the high costs of aP, and its major problem of inducing Th2 immune response are its main drawbacks (16). Therefore, omvPV could be considered as an alternative vaccine types against pertussis (17). Considering the low toxicity of omvPV vaccines and their similarity to wP with respect to stimulation of the immune system, vaccines incorporating OMV from a dominant *Bp* strain of each geographical region could be considered as an alternative solution for the current pertussis dilemma (18). Therefore, the identification of antigenic proteins for a new candidate vaccine is in high demand. The OMV-based vaccines are attractive and useful tools for the complete coverage of vaccination against pertussis while there is still a high prevalence of pertussis among populations of all ages in Iran (19). Here we designed a study to investigate the OMV’s of a vaccine strain and a dominant wild type strains of *Bp* isolates from Iran. Moreover, a quantitative proteomic approach was used to compare the whole-cell vaccine stain 134 with the other regional strains.

In our study, 9 (9%) of infants with PT-IgA were positive for pertussis. Among them, 5 subjects had received 3 doses of wP. The vaccine effectiveness was only estimated to be 33% after receiving 3 doses of wP. Similar low effectiveness results of wP have also been reported in other studies (20). For instance, two trials conducted in Europe, have measured the efficacy of wP, given in 3 primary doses at 2, 4 and 6 months interval. Moreover, Gustafsson and Greco (20, 21) have conducted similar trials in Sweden and Italy, respectively and all of these studies have indicated low effectiveness rates (36%–48.3%) of wP. These have used a particular Connaught vaccine (wP), and it has been suggested that the observed lower efficacies may have been due to the vaccine strain or the poor quality of the vaccine batches. Meanwhile, Blennow and colleagues who have conducted a trial in Sweden using Sauer strains, have found an effectiveness rate of ∼71% (22). However in our study, poor immunogenicity was detected for wP even after administration of 3 doses of the vaccine. However, not all wP vaccines showed equal efficacy, reflecting substantial differences in quality between the manufacturers (23).

The fact that selected DTwP were poorly effective has raised some questions regarding the utility of all DTwP vaccines. Hence, the resurgence of pertussis in Iran may have been contributed to the low effectiveness of wP used. De Greeff et al. results show that, although vaccination programs have reduced pertussis morbidity during the childhood, they have been ineffective to counter the increased infection rate in adolescent and adult pertussis. Indeed, the high circulation of *Bp* in the latter age categories may limit the effectiveness of pediatric vaccination (24). Based on our findings, it seems that a stronger immune response could be observed in children vaccinated with wP, compared with infants infected with the wild-type strain. Waning immunity is regarded as the most significant cause of pertussis resurgence (10). Also, mutations in pertussis toxin as well as pertactin genes are considered as important causes of waning of the vaccine immunity (25). Moreover, prevalence of pertussis outbreaks in adolescents and adults have been revealed even in countries in which the wP or aP vaccination programs have been conducted (19, 26).

Generally, wP induces a complex immune response, due to presence of many bacterial antigens. It induces the production of antibodies against *Bp* key virulence factors, namely, Ptx, FHA, PRN, ACT, LPS, DNT, FIM2/3 and BrkA (16). Raeven et al. (8) have reported that OMV of *Bp* 1917 has 16 protein with significant immunogenic properties. We characterized OMV *Bp* 134 and OMV *Bp* IP91 strains by proteomic analyses and compared their proteome with other strains which had been previously reported (27). Immunoblotting was performed using monoclonal antibodies (i.e. FHA 1:2000, PRN 3:10000 and Ptx 3:1000). According to our results, the candidate antigens for OMV vaccine could be Ptx, FHA and PRN. In addition, antibody responses against *Bp* should preferably be directed against antigens such as FHA, PT and PRN, since they have immune evasive properties (15) and are common in OMV profiles of both the vaccine and the wild type strains.

A comparative analysis of the respective proteomes revealed striking differences between the protein profiles of OMV vaccine and OMV wild type strains. We identified 37 reproducible proteins which had significant changes in their expression levels. A general up-regulation in the proteins levels was observed in OMV IP91 whereas the expression of these proteins levels (namely, those involved in carbohydrate metabolism, lipoprotein biosynthesis and adaptation) were generally down-regulated in OMV 134, due to significant energy cost for OMV production.

Hydrogen-peroxide (H _2_ O _2 _) which has disruptive effects on the metabolism and blocks growth, is commonly generated in biological habitats under defined environmental conditions and even by cellular immune responses. However, some biological reactions lead to detoxification of H2O _2_ . For example, RPE (ribulose-phosphate 3-epimerase) in *E. coli* detoxifies H _2_ O _2_ (28, 29). Interestingly in this study we observed that RPE was overexpressed in wild-type IP91 strain which may protect bacteria against the cellular immune responses.

The low expression of AhpC in the wild type strain shows that the vaccine strain could be more immunogenic than the wild type strain. It has been shown that AhpC is a potent antigen and it induces a promising T cell-mediated immune response in patients with acute Melioidosis (30). The down-regulation of AhpC has been reported in a persistent isolate of *Borkholderia cenocepacia* during a murine infection (31). The antioxidant protein AhpC has been considered as a potential vaccine candidate against various bacterial infections by *Helicobacter pylori, Bacillus anthracis, Streptococcus zooepidemicus* and *Burkholderia pseudomallei* (32–34).

Our results pointed to some other identified outer-membrane proteins such as iron-sulfur cluster carrier protein (*Bp*0182), conserved hypothetical protein (*Bp*3465) and lipoproteins (*Bp*2782 and *Bp*2818). They serve as protective immunogens and are among the principal components of wP vaccines which have been developed against pertussis (27). The function of lipoproteins is unknown, although studies with pathogenic forms of *E. coli* have suggested that they play roles in vesicle formation (35). The lipoproteins from *Bp*2782 and *Bp*2818 which were identified in this study are abound in the outer-membranes of Gram-negative bacteria and for long have been considered as potential target antigens for vaccine development. The immunogenicity of a lipoprotein from the putative outer-membrane of *Bordetella bronchiseptica* has been recently demonstrated (36). Liu et al. have revealed that the recombinant lipoprotein could induce humoral and cell-mediated immune responses in mice (36).

From the 37 identified proteins using LC-MS/MS in current study, 25 proteins including (*Bp* 0103, *Bp* 0205, *Bp* 0558, *Bp* 0627, *Bp* 0777, *Bp* 0961, *Bp* 0962, *Bp* 0965, *Bp* 0995, *Bp* 1126, *Bp* 1300, *Bp* 1487, *Bp* 1616, *Bp* 1857, *Bp* 2360, *Bp* 2361, *Bp* 2434, *Bp* 2533, *Bp* 2533, *Bp* 2770, *Bp* 2818, *Bp* 3228, *Bp* 3277, *Bp* 3552 and *Bp* 3757) were found in other regional study by Tefon (37). *Bp* 2434 (serine protease) and *Bp* 1616 (putative DNA binding protein) were found in all 4 strains studied, including *Bp* Sadat, *Bp* Tahoma I, *Bp* 134 and *Bp* IP91 (37). Serine endoprotease has various effects on the immune response like activation, migration and leukocyte apoptosis according to Ruiz-Perez et al study (38). It has been found that serine protease protein SphB1 is involved in the maturation of FHA in *Bp* (37, 39).

Bacterial DNA binding proteins, also known as histone-like proteins are basic, small proteins which protect DNA in harsh environmental conditions and their immunogenicity has been shown by Altındiş and colleagues (40). During infections, many Gram-negative pathogens secrete their virulence factors via type III secretion system and in *Bordetellae*, this system is active in Bvg+ phase (41). Putative outer protein N (BopN), has been reported as type III secreted protein in *B. bronchiseptica*. In our study, we found that BopN is expressed at a high level in OMV IP91 strain. This indicates that this protein is highly expressed during the natural infection and induces high immune responses.

## CONCLUSION

The shortcomings of wP and aP vaccines, such as their severe side effects and high costs, respectively, have been recently highlighted. OMV VP-based vaccines could be considered as interesting alternative against pertussis. Such complex vaccines which contain the bacterium protein antigens can confer a similar efficacy as wP with lesser side effects and lower costs.
